# Bone marrow lesions detected by specific combination of MRI sequences are associated with severity of osteochondral degeneration

**DOI:** 10.1186/s13075-016-0953-x

**Published:** 2016-02-24

**Authors:** Dzenita Muratovic, Flavia Cicuttini, Anita Wluka, David Findlay, Yuanyuan Wang, Sophia Otto, David Taylor, Julia Humphries, Yearin Lee, Agatha Labrinidis, Ruth Williams, Julia Kuliwaba

**Affiliations:** Discipline of Orthopaedics and Trauma, The University of Adelaide, Adelaide, Australia; Bone and Joint Research Laboratory, SA Pathology, Frome Road, Adelaide, 5000 Australia; Department of Epidemiology & Preventive Medicine, Monash University, Melbourne, Australia; Anatomical Pathology, SA Pathology, Adelaide, Australia; Department of Radiology, Royal Adelaide Hospital, Adelaide, Australia; Adelaide Microscopy, The University of Adelaide, Adelaide, Australia

**Keywords:** Knee osteoarthritis, Bone marrow lesion, MRI, Osteochondral unit, Subchondral bone, Cartilage

## Abstract

**Background:**

Bone marrow lesions (BMLs) are useful diagnostic and prognostic markers in knee osteoarthritis (OA), but what they represent at the tissue level remains unclear. The aim of this study was to provide comprehensive tissue characterization of BMLs detected using two specific MRI sequences.

**Methods:**

Tibial plateaus were obtained from 60 patients (29 females, 31 males), undergoing knee arthroplasty for OA. To identify BMLs, MRI was performed *ex vivo* using T1 and PDFS-weighted sequences. Multi-modal tissue level analyses of the osteochondral unit (OCU) were performed, including cartilage volume measurement, OARSI grading, micro-CT analysis of bone microstructure, routine histopathological assessment and quantitation of bone turnover indices.

**Results:**

BMLs were detected in 74 % of tibial plateaus, the remainder comprising a No BML group. Of all BMLs, 59 % were designated BML 1 (detected only by PDFS) and 41 % were designated BML 2 (detected by both PDFS + T1). The presence of a BML was related to degeneration of the OCU, particularly within BML 2. When compared to No BML, BML 2 showed reduced cartilage volume (p = 0.008), higher OARSI scores (p = 0.004), thicker subchondral plate (p = 0.002), increased trabecular bone volume and plate-like structure (p = 0.0004), increased osteoid volume (p = 0.002) and thickness (p = 0.003), more bone marrow oedema (p = 0.03), fibrosis (p = 0.002), necrosis (p = 0.01) and fibrovascular cysts (p = 0.04). For most measures, BML 1 was intermediate between No BML and BML 2.

**Conclusions:**

BMLs detected by specific MRI sequences identify different degrees of degeneration in the OCU. This suggests that MRI characteristics of BMLs may enable identification of different BML phenotypes and help target novel approaches to treatment and prevention of OA.

## Background

Knee osteoarthritis (OA), a painful degenerative condition with no effective treatment, is one of the leading causes of human suffering. It is widely accepted that OA is a disease of the whole joint, with particular involvement of the articular cartilage and subchondral bone. In fact, these two tissues act as a functional unit, the osteochondral unit (OCU), to maintain joint homeostasis [1]. Pathological changes in either the bone or the cartilage seem to predict degenerative changes in the other.

Focal changes in the subchondral bone, termed bone marrow lesions (BMLs), are features detected by magnetic resonance imaging (MRI) that have been reported to be closely associated with the severity of symptoms of OA such as pain [2–4] and OCU degeneration (e.g., loss of the overlying cartilage) [5–8]. Clinical studies have reported BMLs in both patients with early asymptomatic OA [9–12] and in those with severe late-stage OA [6, 13–15]. In patients with early OA and in individuals who do not have OA, BMLs can decrease in size or resolve completely [2, 5, 16]. Hunter et al. reported that in progressive OA, BMLs are more likely to persist and to enlarge in size [6]. Previous human histological studies examined small numbers of samples and found mixed pathological findings of bone marrow and sclerotic bone in BMLs [17–19]. Similar histopathological findings have been reported for animal models of OA [20].

BMLs are conventionally assessed using fat-suppressed or proton-dense T2-weighted MRI, although they may also be detected using other MRI sequences. Within fat-suppressed T2-weighted and/or proton density-weighted sequences they appear as areas of ill-defined hyperintensity (high signal) in subchondral bone, and in T1-weighted sequences they appear hypointense (low signal) [21–25]. Thus, although fat-suppressed T2-weighted and/or proton density-weighted sequences are recommended for the assessment of BMLs as they depict lesions to their maximum extent, T1-weighted sequences are predominantly used for assessment of the cartilage. Preferably, a combination of sequences should be used to evaluate the extent of OA disease progression [5, 14, 26, 27].

There is extensive debate about the optimal way to image BMLs but it remains unknown whether BMLs detected by different MRI sequences differ at the tissue level. Thus, it is possible that amongst BMLs identified by conventional T2-weighted images, some may also be detectable using another MRI sequence, but others may not. This would suggest that the underlying tissues in these groups are not the same and may thus relate to different clinical outcomes. As BMLs are closely associated with pain and loss of cartilage [12, 14, 28, 29], they are emerging as promising targets for monitoring progression of knee OA [30] and the effects of treatment [31]. Therefore, a comprehensive understanding of the underlying pathology of BMLs is important.

The aim of this study was to comprehensively investigate histological changes in all components of the OCU (cartilage, subchondral bone and subchondral bone marrow) based on the presence or absence of a BML detected by two specific MRI sequences, in tibial plateau tissue obtained during knee replacement surgery.

## Methods

### Patient samples

Tibial plateaus (TP) were obtained from 60 patients undergoing knee arthroplasty surgery (29 female patients aged 51 to 87 years, body mass index (BMI) range 24.1–41.4 and 31 male patients aged 42 to 86 years, BMI range 22.6–45.7). Written consent was obtained from all patients and the study received prior approval from the Human Research Ethics Committee at the Repatriation General Hospital, Royal Adelaide Hospital and The University of Adelaide, South Australia, in accordance with the Declaration of Helsinki 1975.

Inclusion criteria were: radiographic evidence of OA with severe symptomatic disabilities, such as severe pain and limited mobility. Exclusion criteria were secondary OA of the knee due to trauma or rheumatoid arthritis, evidence of bone-related chronic debilitating disease and/or history of any medication that may have affected bone turnover.

### Macroscopic evaluation

All retrieved TP were examined and graded macroscopically according to the Outerbridge Classification [26, 32] by two experienced orthopedic surgeons (DM and CW), for whom the intraclass correlation coefficient (ICC) for inter-observer reproducibility was 0.81 (95 % CI 0.79, 0.84).

### Radiographic evaluation of knee OA

Standing anteroposterior, posteroanterior and lateral projection radiographs were taken prior to surgery. The extent of radiographic evidence of progression of OA was assessed according to the Kellgren and Lawrence (K&L) grade, the current standard radiologic grading system for OA. All radiographs were scored by two experienced assessors (AW and YW) with 5 % disagreement. Assessors were blinded to the presence of BMLs in the knee joint.

### Magnetic resonance imaging

Specimens were scanned ex vivo using an MRI scanner with an 8-channel wrist coil (3 T MRI Siemens TRIO, Berlin, Germany), with two specific sequences: fat-suppressed (FS) fast spin-echo proton density-weighted (PDFS) and T1-weighted spin echo in the sagittal and coronal planes. Sagittal slice thickness was 1.6 mm with a distance factor of 25 %; coronal slice thickness was 3.0 mm with a 10 % distance factor. We confirmed that ex vivo MRI information corresponded to pre-operative imaging, by comparing pre- and post-operative MRI data for a subset of five patients, consistent with previous reports [20, 33, 34].

**Fig. 1:**
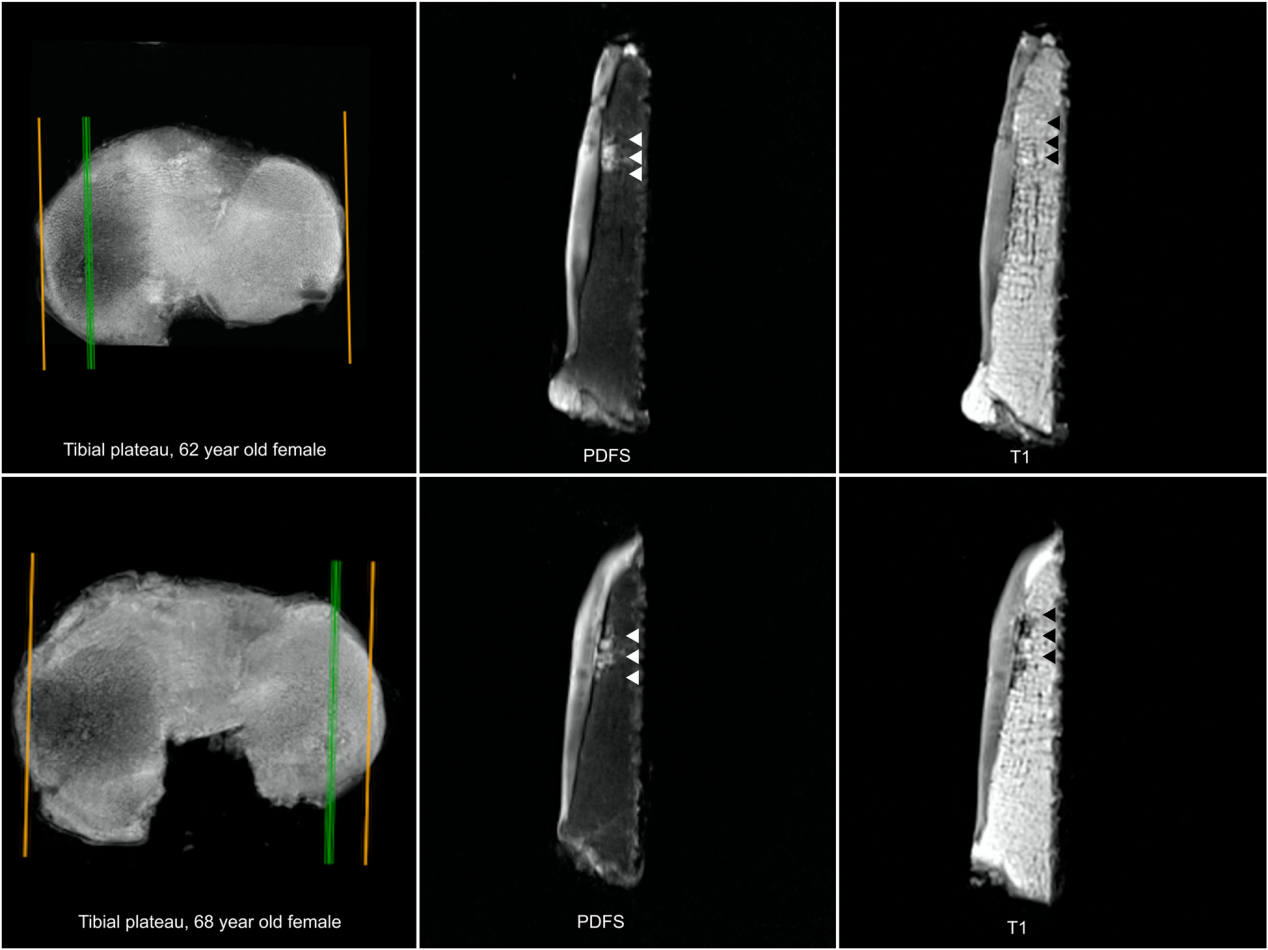
*Left*, *upper* and *lower panels* show tibial plateaus in the transaxial plane. *Right*, *green line* is the sagittal slice. *Upper panel*, *middle*, the signal of the bone marrow lesion (BML) is detected by fast spin-echo proton density-weighted (PDFS) sequence (*arrows* identify signal), and *right* the signal is not detected by T1-weighted sequence. *Lower panel*, *middle*, the signal of the BML is detected by PDFS sequence (*arrows* identify signal), and *right* the signal is detected by T1-weighted sequence

The definition for identification of BMLs was by mutual agreement between two radiologists with musculoskeletal MRI expertise (DT and YW). A BML is defined as a zone of altered signal intensity in the bone and marrow, located immediately beneath the articular cartilage and visible on at least two consecutive slices [7, 13, 35]. Based on the presence and/or absence of signal, two subtypes of BML were defined. BMLs detected using the PDFS sequence only and with absent signal on T1-weighted sequence in the same area are referred to as BML 1; BMLs detected by both PDFS and T1 sequences are referred to as BML 2. (Fig. 1 shows examples of BML 1 and BML 2).

**Fig. 2:**
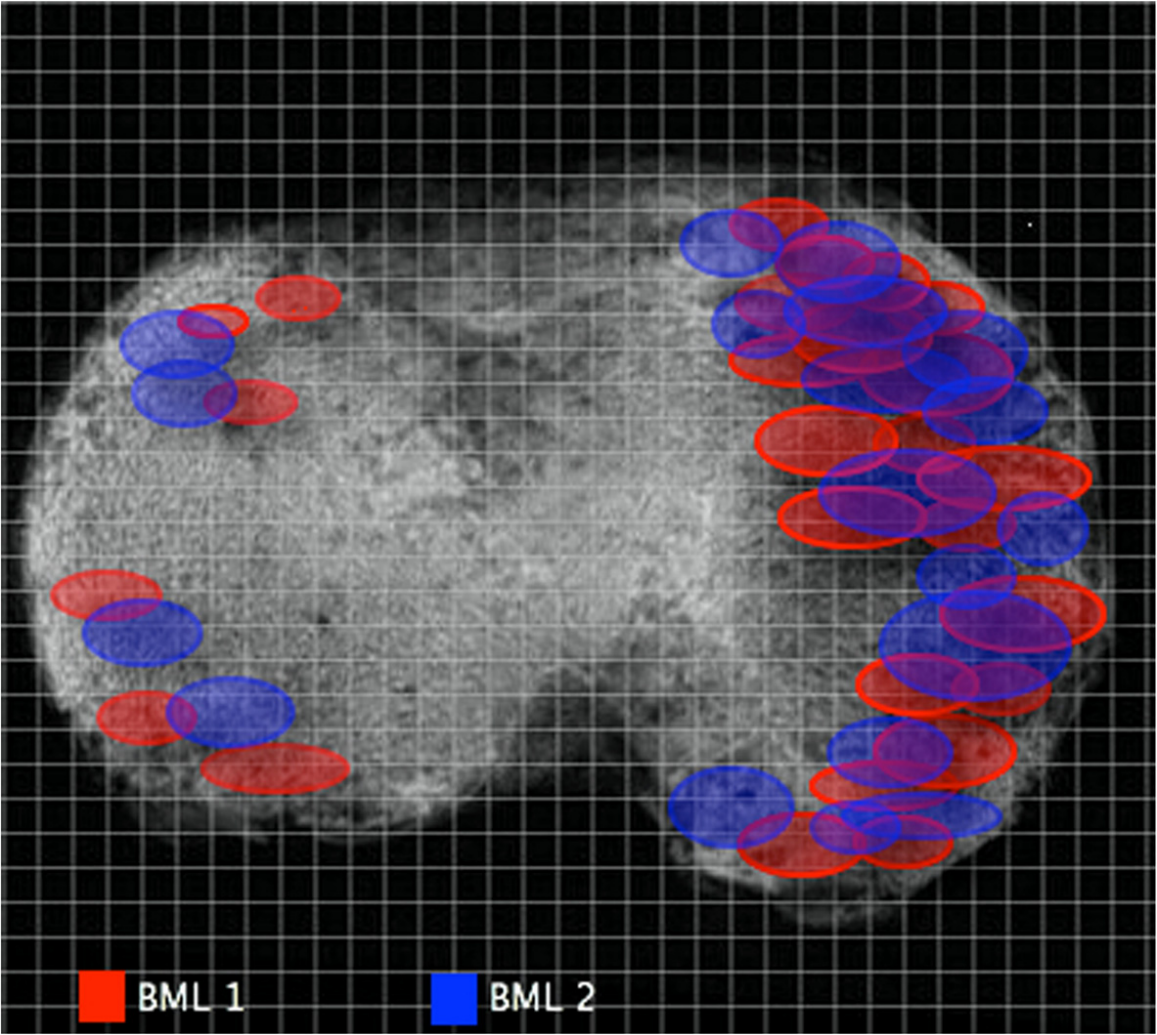
The approximate external contour of each bone marrow lesion (*BML*) area was marked. The precise map location was placed by identifying the number of sagittal and coronal slices with measurement of distance from the external tibial contour. After marking the position of the BML for all specimens, a distribution map of both types of BML was found. *BML 1* bone marrow lesion detected using the fast spin-echo proton density-weighted sequence only, with absent signal on T1-weighted sequence in the same area, *BML 2* bone marrow lesions detected by both fast spin-echo proton density-weighted and T1 sequences

After identification of a BML the external contours of the BMLs were marked in both planes by two researchers (DzM and YRL) blinded to the presence of BMLs. The volume and precise location of each BML was determined, enabling the creation of a two-dimensional (2D) axial map of all BMLs (Fig. 2 demonstrates the approximate size and location of both BMLs). Cartilage volume in the medial compartment was determined as described previously [7, 25]. The coefficient of variation for the measurement of cartilage volume at the medial tibia was 2.2 %.

### Micro-computed tomography (micro-CT)

A subset of 36 TP (6 mm minimal thickness) was scanned by micro-CT (Skyscan model 1076, Kontic, Belgium). Images were obtained at isotropic resolution of 17.4 μm. Using AVIZO® Fire software (Zuse Institute, Berlin, Germany), and three-dimensional (3D) volumes of TP were generated using both MRI and micro-CT images. Then, the BML signal location was identified from MRI and aligned onto the 3D volume of micro-CT. A cylindrical region of interest (ROI) containing the volume of the BML, diameter 10 mm × depth 6 mm, was used for analysis of BML bone microstructure. As BMLs were found predominantly on the medial side, the same size and shape ROI was used in the medial compartment of the TP without BMLs. These ROIs were further divided into the subchondral plate and subchondral trabecular bone regions and analyzed separately, using CT-An analyzer software (SkyScan).

### Microscopic evaluation

A cuboidal block of cartilage-subchondral bone (10 × 10 × 5 mm), representing the area containing a BML (named BML), was dissected from the TP using a low-speed diamond saw (Model 660, South Bay Technology, San Clemente, US). A tissue block of the same size and shape was cut from the medial compartment of the TP without BMLs (no BML). Each cube was divided equally, with one half formalin-fixed, processed and embedded in methyl-methacrylate resin. The block was cut into 5-μm-thick sections and stained with von Kossa silver/hematoxylin and eosin (H&E) for histomorphometric analysis of bone remodeling. The other half of the block was formalin-fixed, decalcified in 5 % hydrochloric acid, paraffin-embedded, sectioned 5-μm-thick and stained with H&E and Safranin-O/Fast Green. A senior pathologist (SO) with over 10 years of experience in the field, blinded to the MRI findings, used a 1–5 scoring system to semiquantitatively evaluate the presence and extent of pathological findings in the tissue on the H&E slides, where 1 = <5 % (minimal presence), 2 = 5–14 %, 3 = 15–25 % (moderate presence), 4 = 25–50 % and 5 = >50 % (prominent presence). The intra-observer reproducibility of the histological scores (assessed by SO) was measured at separate times for ten sections (ICC 0.98 (95 % CI 0.95, 0.98)). Safranin-O/Fast Green was used for Osteoarthritis Research Society International (OARSI) grading [36, 37]. Consensus between three assessors (DzM, EG and YRL) determined the grading. The ICC for inter-observer reproducibility was 0.82 (95 % CI 0.80, 0.84).

### Statistical analysis

The Shapiro–Wilk test was used to determine normality of the data distribution. Differences between the no BML and the BML (BML 1 + BML 2) groups were described using the unpaired *t* test for parametric distribution or the Mann–Whitney *U* test for non-parametric data distribution. Differences between three groups (no BML (no BML detected), BML 1 (BML detected only by PDFS sequence) and BML 2 (BML detected by both PDFS and T1 sequences)) were described using analysis of variance (ANOVA). For parametric data, ANOVA and the Holm–Sidak comparison test with single pooled variance were performed. For non-parametric data, the Kruskal–Wallis test and Dunn’s multiple comparison test were performed. An adjusted model was then performed for all outcome parameters versus BML group, adjusting for age, sex and BMI. *P* values <0.05 were considered to be statistically significant.

## Results

Demographic characteristics of the participating individuals were grouped according to the presence or absence of BML on specific MRI sequences, and are summarized in Table 1. There were no significant differences between the two groups in patient age, gender, BMI or K&L grade.

**Table 1:** Patient demographic characteristics

	No BML (n = 12)	Total BML (1 + 2) (n = 44)	*P* value
Age^a^	69.8 ± 1.4	68.3 ± 1.2	0.5
Male, number (%)	3 (25 %)	25 (57 %)	0.06
Female, number (%)	9 (75 %)	19 (43 %)	0.1
BMI^a^	31.3 ± 6.4	33.4 ± 5.1	0.2
K&L grade^b^	2 (1, 4)	3 (2, 4)	0.7
Medial OA, number of patients (%)	6 (50 %)	34 (77 %)	0.07
Lateral OA, number of patients (%)	2 (16 %)	6 (14 %)	0.5
Patello-femoral OA, number of patients (%)	4 (33 %)	4 (9 %)	*0.04*
Subchondral cyst present, number of patients (%)	0 (0 %)	12 (27 %)	*0.03*
Outerbridge classification^b^	3 (3, 4)	4 (3, 4)	*0.04*
Cartilage volume^a^	1.3 ± 0.2	0.9 ± 0.3	*0.01*
OARSI score^a^	3.7 ± 1.2	5 ± 0.9	*0.004*

^a^Values presented with mean ± standard deviation. ^b^Values presented with median (25^th^, 75th percentiles). *P* values are for difference between the group with no bone marrow lesions (No BML) and the group with BML (BML 1 + BML 2). *BMI* body mass index, *K&L* Kellgren and Lawrence, *OA* osteoarthritis, *OARSI* Osteoarthritis Research Society International

BMLs were detected in 44 (73 %) of TP; 12 (20 %) of TP were without BML and/or subchondral cysts (the no BML group). Of the TP with a BML, 12 (27 %) also had a subchondral cyst present in the intercondylar space. Furthermore, 4 TP (6 % of all subjects) had cysts but without BML and therefore were excluded from further analysis. BMLs detected using the PDFS sequence only (BML 1) represented 59 % of all BMLs. The signal intensity in these lesions was either moderate or diffuse in the PDFS sequence and by definition there was no signal on the T1-weighted sequence in the same areas. BMLs detected by both PDFS and T1 sequences (BML 2) represented 41 % of all BMLs. The signal intensity in BML 2 was hyperintense on the PDFS sequence and hypointense on the T1-weighted sequence. Preoperative radiographs indicated that 77 % of TP with BML were diagnosed with medial OA, 14 % with lateral OA and 9 % with patellofemoral OA (Table 1). Furthermore, both BML types were present predominantly in the medial compartment of TP (87 %), with their anatomical distribution aligning closely with the menisci (Fig. 2).

Firstly, we examined whether structural changes in all components of the OCU (cartilage, subchondral bone and subchondral bone marrow) differed based on the presence or absence of a BML detected by two specific MRI sequences. In TP with BML, areas corresponding to a BML, either BML 1 or BML 2, were compared to anatomically matched areas in TP without BML, and progressive degenerative changes were found in BML areas for all tested parameters. These included a higher Outerbridge score, reduced cartilage volume, higher OARSI score (Table 1), more histopathological abnormalities such as tidemark duplication, penetration of vascular cones into calcified cartilage, edema, necrosis, fibrosis, the presence of thick-walled arterioles, and small fibrovascular cystic formations (Table 2). BML containing subchondral bone had thicker subchondral plate, increased trabecular bone volume, more trabeculae that were predominantly plate-like, increased osteoid volume and thickness of both plate and trabeculae and decreased eroded surface in trabecular bone (Table 3).

**Table 2:** Histological findings in the no BML and BML groups

	No BML (n = 12)	BML (1 + 2) (n = 44)	*P* value
TM, number/mm^2 a^	2 (2, 3)	3 (3, 4)	*<0.0001*
Vascular cones, number/mm^2 b^	0.12 ± 0.07	0.20 ± 0.12	*0.004*
Edema			*0.006*
Score 1 (<5 %)	83 %	41 %	
Score 2 (5–14 %)	17 %	31.8 %	
Score 3 (15–24 %)	-	20.4 %	
Score 4 (25–50 %)	-	6.8 %	
Score 5 (>50 %)	-	-	
Fibrosis			0.4
Score 1 (<5 %)	75 %	66 %	
Score 2 (5–14 %)	25 %	16 %	
Score 3 (15–24 %)	-	11 %	
Score 4 (25–50 %)	-	7 %	
Score 5 (>50 %)	-	-	
Necrosis			*0.002*
Score 1 (<5 %)	33.1 %	9 %	
Score 2 (5–14 %)	66.6 %	41 %	
Score 3 (15–24 %)	-	41 %	
Score 4 (25–50 %)	-	6.8 %	
Score 5 (>50 %)	-	2.2 %	
Th. wall Art., number/mm^2 a^	0.07 (0.06, 0.1)	0.2 (0.1, 0.4)	*<0.0001*
Fibrovascular c., number/mm^2 a^	0 (0 %)	0 (0, 0.2)	*0.01*

^a^Values presented with median (25^th^, 75th percentiles). ^b^Values presented with mean ± standard deviation. *P* values are for difference between the group with no bone marrow lesions (No BML) and the group with BML (BML 1 + BML 2). *TM* tidemark duplications, *Vascular cones* vascular cones penetrating from subchondral bone into calcified cartilage, *Th. wall Art* thick-walled arterioles present in marrow, *Fibrovascular c*. fibrovascular cystic formations

**Table 3:** Micro-CT analysis and bone turnover

	No BML (n = 12)	BML (1 + 2) (n = 44)	*P* value
Micro-CT - subchondral plate			
Pl.Th, mm^a^	0.44 ± 0.10	0.95 ± 0.29	*0.001*
Surface cl.pores^b^	49.5 (23, 83.7)	103.7 (60, 203)	*0.007*
Micro-CT - subchondral trabeculae			
BV/TV, %^a^	12.06 ± 3.5	16.7 ± 5.1	*0.02*
Tb.N, number/mm^a^	0.9 ± 0.2	1.2 ± 0.3	*0.04*
Tb.Sp, mm^b^	0.9 (0.9, 1)	1.2 (1, 1.5)	*0.06*
Tb.Th, mm^b^	0.13 (0.11, 0.15)	0.14 (0.12, 0.16)	0.2
Tb.Pf^a^	-2.2 ± 3.7	-9.3 ± 7.8	*0.02*
SMI^a^	1.32 ± 0.4	0.62 ± 0.8	*0.02*
DA^b^	1.5 (1.4, 1.6)	1.6 (1.5, 1.8)	0.5
Bone remodeling - subchondral plate			
O.Th, μm^a^	36 ± 8.9	43 ± 10.9	*0.03*
OS/BS, %^b^	2.5 (1.1, 3.6)	5.1 (3.6, 7.2)	*0.0001*
OS/BV, mm^2^/mm^3b^	1.9 (0.8, 2.8)	2 (1.5, 3.6)	0.3
OS/TV, mm^2^/mm^3a^	0.4 ± 0.2	0.8 ± 0.4	*0.004*
OV/BV, %^b^	7.1 (3.4, 8.8)	9.4 (6.1, 15)	*0.08*
OV/TV, %^b^	1.6 (0.7, 2.1)	3.5 (2, 5.6	*0.001*
ES/BS, %^b^	0.03 (0, 0.1)	0.09 (0.01, 0.17)	0.1
ES/BV, mm^2^/mm^3b^	0.02 (0, 0.06)	0.03 (0.007, 0.07)	0.5
ES/TV, mm^2^/mm^3b^	0.004 (0, 0.01)	0.01 (0.002, 0.02)	0.1
Bone remodeling - subchondral trabeculae			
O.Th, μm^b^	24.7 (22.4, 32.2)	38.1 (27.6, 46.3)	*0.005*
OS/BS (%)^b^	3.4 (1.9, 3.9)	3 (1.4, 5.8)	0.6
OS/BV, mm^2^/mm^3b^	1.9 (1.1, 2.9)	2.2 (1.4, 3.5)	0.3
OS/TV, mm^2^/mm^3b^	0.4 (0.3, 0.5)	0.7 (0.4, 1.1)	*0.04*
OV/BV, %^b^	3.6 (2.3, 5.5)	4.7 (2.0, 7.3)	*0.04*
OV/TV, %^b^	1.2 (0.8, 1.7)	2 (1.2, 5.4)	*0.008*
ES/BS, %^b^	0.2 (0.1, 0.5)	0.1 (0.09, 0.2)	*0.02*
ES/BV, mm^2^/mm^3b^	0.1 (0.04, 0.1)	0.07 (0.05, 0.1)	0.8
ES/TV, mm^2^/mm^3b^	0.02 (0.01, 0.03)	0.02 (0.01, 0.04)	0.7

^a^Values presented with mean ± standard deviation. ^b^Values presented with median (25^th^, 75th percentiles). *P* values are for difference between the group with no bone marrow lesions (no BML) and the group with BML (BML 1 + BML 2). *cl.pores* closed pores, *Micro-CT* micro computed tomography, *BV* bone volume, *TV* tissue volume, *Tb.N* trabecular number, *Tb.Sp* trabecular separation, *SMI* structural model index, *Tb.Th* trabecular thickness, *O.Th* osteoid thickness, *OS* osteoid surface, *BS* bone surface, *ES* erosion surface, *OV* osteoid volume, *Pl.Th *Plate thickness, *Tb.Pf* trabecular patern factor, *DA *degree of anisotropy

**Fig. 3:**
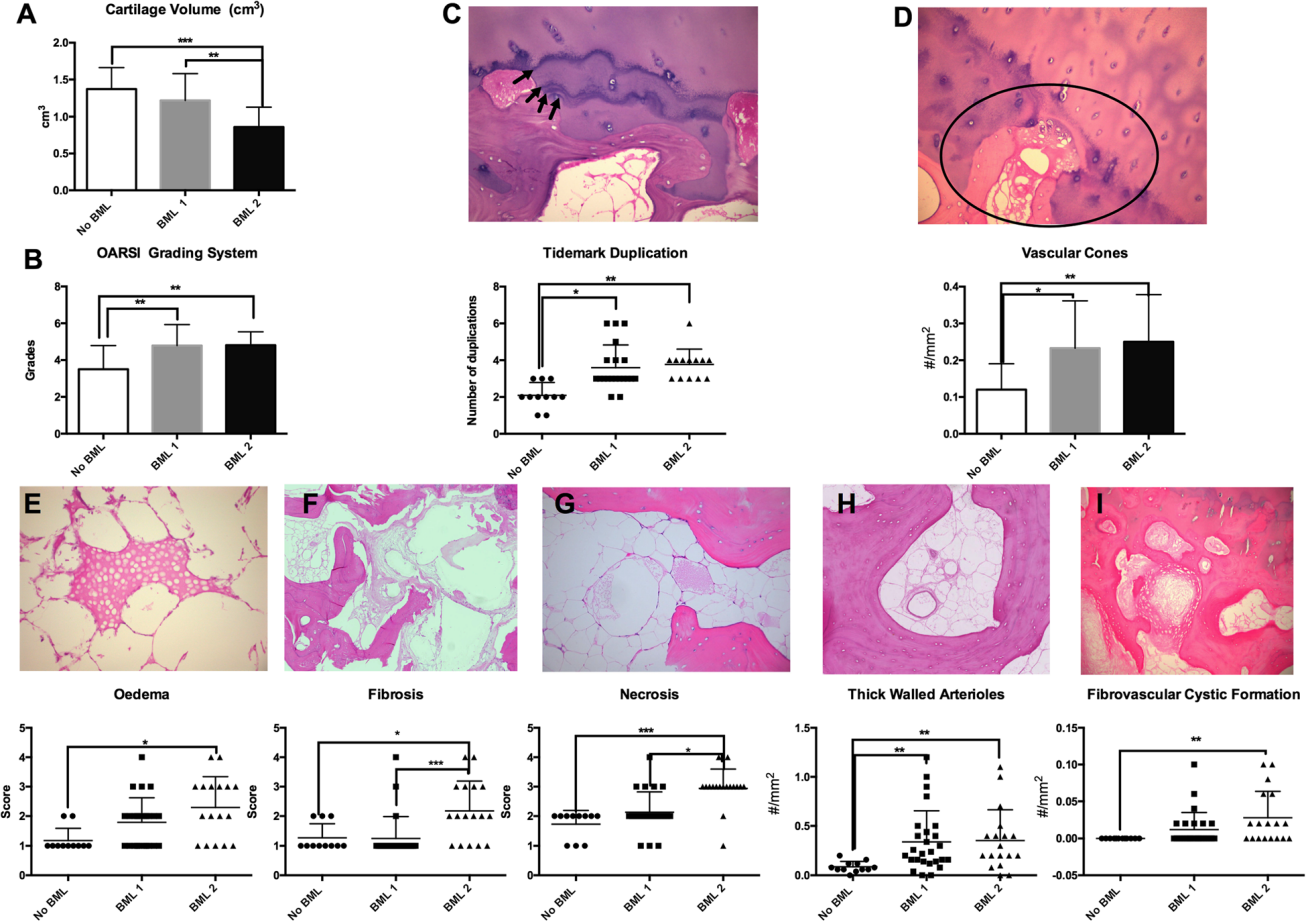
Comparison of cartilage volume (**a**), Osteoarthritis Research Society International; (OARSI) grades (**b**), cartilage tidemark progression (*arrows* identify tidemarks) (**c**), penetration of vascular cones (*circled*) (**d**). Histopathology of bone marrow; bone marrow edema (**e**), fibrosis (**f**), necrosis (**g**), thick-walled arterioles (**h**) and fibrovascular cystic formation (**i**). Represented histologically and graphically for specimens with no bone marrow lesions evident (*No BML*), bone marrow lesions evident only in fast spin-echo proton density-weighted (PDFS) sequences (*BML 1*), and bone marrow lesions evident in both PDFS and T1-weighted sequences (*BML 2*): *p = 0.05, **p = 0.005, ***p = 0.0005

**Fig. 4:**
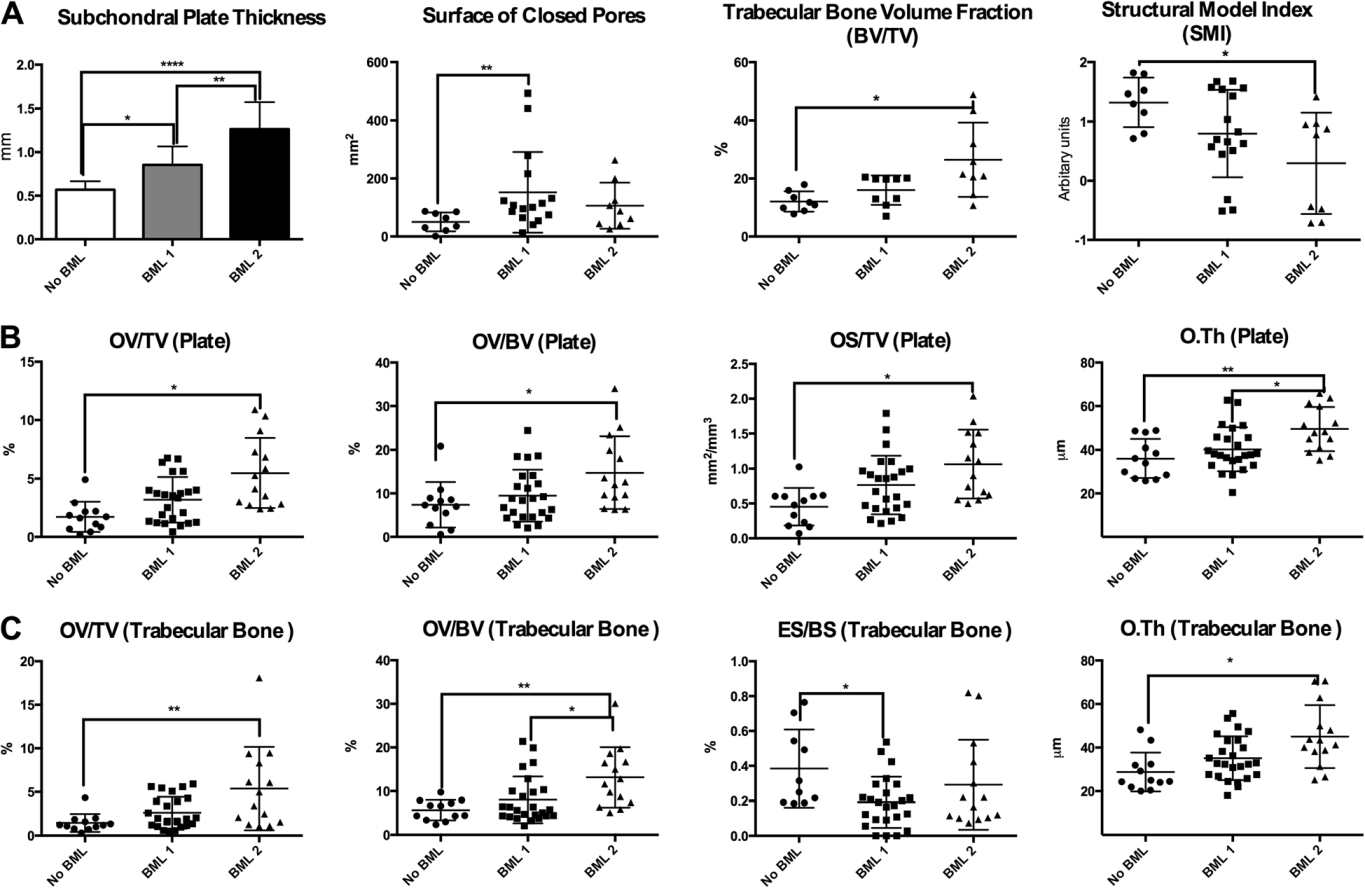
Micro-computed tomography bone analysis (**a**), subchondral plate, thickness and surface of closed pores; subchondral trabecular bone, bone volume fraction and structural model index. Bone turnover in subchondral plate (**b**), osteoid volume over tissue volume, volume of osteoid, osteoid surface over tissue volume and thickness of osteoid. Bone turnover in trabecular bone (**c**), osteoid volume over tissue volume, volume of osteoid, erosion surface over bone surface and thickness of osteoid. Represented graphically for specimens with no bone marrow lesions evident (No BML), bone marrow lesions evident only in fast spin-echo proton density-weighted (PDFS) sequences (BML 1), and bone marrow lesions evident in both PDFS and T1-weighted sequences (BML 2): *p = 0.05, **p = 0.005, ***p = 0.0005. *BV* bone volume, *TV* tissue volume, *OV* osteoid volume, OS osteoid surface, *O.Th* osteoid thickness, *ES* erosion surface, *BS* bone surface

We then examined whether the extent of these changes was different depending on the BML subtype. BMLs detected by both PDFS and T1 sequences (BML 2) were identified as lesions having the most advanced degenerative changes throughout the whole OCU. In fact, they displayed all the changes described above for subchondral bone with BML signal (Figs. 3 and 4). In contrast, BMLs detected only by the PDFS sequence (BML 1) displayed a subset of the intermediate degenerative changes when compared to TP with no BML and those with BML 2 (Figs. 3 and 4).

To assess whether the histological composition of BML visualized by different sequences differed, we compared BML 1 and BML 2. The BML 2 were associated with reduced cartilage volume (*p* = 0.007) more fibrosis (*p* = 0.006) and necrosis (*p* = 0.01) in the bone marrow (Fig. 3), thicker subchondral bone plate (*p* = 0.002), with higher osteoid thickness (O.Th) (*p* = 0.04) in trabecular bone and with no differences between histomorphometric parameters besides higher osteoid volume/bone volume (OV/BV) (*p* = 0.02) compared to BML 1 (Fig. 4).

## Discussion

In this study we found that BMLs detected using different MRI sequences in OA can differentiate the degree of degeneration through the OCU. Broadly, two subtypes of BMLs could were identified by specific MRI sequences, which corresponded to different tissue features. BML 1, seen only on the PDFS sequence involved less severe OA change than BML 2, seen on both PDFS and T1 sequences.

Our study confirmed previous findings of BMLs being predominantly present in the medial compartment of the tibial plateau. Importantly, we found that loss of cartilage volume and degenerative changes were strongly influenced by the presence of a specific type of BML. The tibial cartilage volume was found to decrease progressively in the knee with no BML, to BML 1 (detected using the PDFS sequence only), and to be almost completely lost with BML 2 (BMLs detected by both PDFS and T1 sequences). The loss of medial tibial cartilage overlying BMLs is consistent with previous clinical studies [7, 8, 38] showing that the presence of BMLs in the medial compartment increases the risk of radiographic evidence of progression of OA (hazard ratio 6.5) and greater cartilage volume loss, [39]. Our finding of BML-related loss of cartilage supports the notion that changes in the subchondral bone are connected to the progression of OA and that it is of significant importance to increase our understanding of BMLs.

Our findings of histological differences between BMLs detected by different MRI sequences are intriguing. The study by Zanetti et al. was one of the first to describe the histology of BMLs and in that study it was found that edema is minimally present, suggesting the term “bone marrow lesion” is used for these features [17]. Our results showed that BML 2 had significantly greater edema, fibrosis and necrosis present in bone marrow compared to the no BML group and BML 1. Other human and animal histological studies [18–20] confirmed that BMLs are characterized by mixed pathological appearances and have not found specific histopathological changes in BML to explain the MRI signal, although it was suggested by Saadat et al. that the hyperintense MRI signal might result from increased blood flow [40]. We analyzed the density of vascular cones in the subchondral plate and the number of small thick-walled arterioles in the bone marrow corresponding to the BML signal in ex vivo samples. We found that the subchondral plate and the marrow of both BML 1 and BML 2 contained significantly more vascular cones and small thick-walled arterioles compared to the no BML group. Therefore, although there was obviously no blood flow in the ex vivo samples, it remains possible that the signals relate somehow to the altered vascular structures associated with BMLs.

Guymer et al. found that the presence of BMLs is closely associated with high BMI and suggested that obesity might be an important factor in their formation [10]. Similarly, Felson et al. found that knee malalignment and high loading of the joint associate with the presence of BMLs [28]. In this study, we did not find a significant relationship between BMI and the presence of BMLs but we did not measure knee malalignment. It is likely that knee loading is not simply a function of BMI, but involves both the frequency and manner of loading. By creating a distribution map of lesions in the tibial plateau, we found that both types of BML are predominantly located in the medial compartment, which is most commonly affected by degenerative changes in knee OA. Furthermore, contours of the BML distribution correspond to the contours of the tibial areas with high contact pressure [41]. Several studies have suggested that high repetitive joint loading can initiate a cellular response to bone injury and/or microdamage in terms of increased bone remodeling. Increased remodeling is closely associated with thickening of the subchondral plate (endochondral ossification evident as presence of multiple tidemark duplications) and increased vascular invasion of the deep layers of articular cartilage, which leads to loss of cartilage thickness and integrity, and finally complete cartilage loss. Subchondral bone in OA undergoes sclerotic changes, with increased trabecular number and volume but with decreased mineralization [15, 42–44]. Although less studied, the subchondral plate also shows thickening [45, 46], which is believed to increase with progression of OA [19, 43, 47, 48]. The present study found each of those phenomena to be highly represented within the tibial plateau with BML 2, intermediate with BML 1 and minimal with no BML, supporting a mechanical origin of BMLs.

Interestingly, BML 2 bone displayed increased osteoid thickness in the subchondral plate and increased osteoid volume in the trabeculae, compared to BML 1. This may indicate a defect in osteoid mineralization, previously linked with increased levels of transforming growth factor (TGF)-β in OA, which is reported to occur prior to loss of cartilage [49]. A recent study by Zhen et al*.* in mice suggests an important role of TGF-β1 in both subchondral pathological change and cartilage degeneration during the progression of OA [50]. Evidence was obtained from transgenic animals over- or under-expressing TGF-β1 to show that high concentrations of TGF-β1 in the subchondral bone contribute to OA development and progression. Similar to the present findings in human OA BMLs, increased osteoid and vasculature were consequences of TGF-β over-expression. The authors suggested that the osteoid islets might correspond to the BML signal detected by MRI [50]. Although this is possible, we have not been able to unequivocally assign the BML signals to any specific feature in the subchondral bone.

Our data suggest that the use of specific MRI sequences offers potential application for OA disease staging and to identify individuals with more advanced structural progression of disease. In particular, BML 2 appears to represent subchondral tissue and cartilage with more degenerative structural changes and therefore less ability to resolve or repair (Table 4). As we found intermediate differences between BML 1 and BML 2, we propose that BML 1 might be an early or transitional stage of BML. In both human and animal studies it has been found that BMLs in the early stage of disease are dynamic and can resolve within time. Perhaps BMLs seen only by PDFS MRI sequences are those that have the ability to resolve [16, 51], making them a potential target for early diagnosis and potential therapy. Further studies of early-stage OA are needed to confirm this possibility and to investigate modifiable risk factors for the initiation of BMLs. The importance of BMLs as therapeutic targets has been recognized, and there are current studies in which BML size and frequency are serving as an outcome measure [39, 52]. Therapies that target BMLs as biomarkers of the initiation and/or progression of knee OA might be more effective than those targeting cartilage repair, as cartilage degradation might be a consequence of failed repair mechanisms in subchondral bone and/or bone marrow. We therefore propose that BML 1 may be a better candidate for targeted treatments and as an outcome measure, than BML 2.

**Table 4:** Group with no bone marrow lesions (No BML) vs*.* groups with BML 1 and BML 2

	No BML vs. BML 1	No BML vs. BML 2
Cartilage		
MRI cartilage volume	Not different	Low
OARSI histology score	High	High
Bone marrow pathology		
Edema	Not different	High
Fibrosis	Not different	High
Necrosis	Not different	High
Vascularity	High	High
Subchondral bone		
Plate thickness	High	Very high
Trabecular bone volume	Not different	High
Osteoid volume and thickness	Not different	High

Changes at tissue level between the No BML group vs*.* group with BML detected using the fast spin-echo proton density-weighted sequence only, with absent signal on T1-weighted sequence in the same area (BML 1) and the group with BML detected by both fast spin-echo proton density-weighted and T1 sequences (BML 2). *MRI* magnetic resonance imaging, *OARSI* Osteoarthritis Research Society International

This study has several limitations. First, we have only examined ex vivo tibial plateau samples from patients with advanced and painful knee OA. However, this limitation will be present for any human OA histopathological studies. It may be important that our recent clinical study broadly supports our ex vivo findings, namely that BML 2 was associated with greater cartilage volume loss and more incident pain [38]. Second, BMLs were identified post-operatively and damage during handling could possibly have led to altered signal on the post-operative MRI. We have taken care to minimize potential artifacts in post-operative MRI by using the same handling protocol for all specimens and by excluding the cut surfaces from our analysis. Third, we only investigated BMLs from knee OA and findings might differ for other skeletal joints. We believe that the strength of this study is that we have analyzed a large number of specimens compared to previous studies, using a comprehensive multi-modal analysis of changes in cartilage, bone and bone marrow in association with BMLs. Fourth, the thickness of our specimens was between 5 and 15 mm, and clinically BMLs may be considerably larger than this and might expand to a greater depth within the tibia. On the other hand, it has been found that bone structural changes are most prominent in the first 6 mm of depth beneath the cartilage [42].

## Conclusion

The presence of BMLs detected by specific MRI sequences is strongly associated with the degree of structural change in the OCU in knee OA. Furthermore, different MRI sequences appear able to differentiate different degrees of structural damage in knee OA. Therefore, BMLs detected with specific sequences could act as potential MRI biomarkers for the identification of individuals at high risk of progressive OA or for development and monitoring of new therapies for this condition.

## Abbreviations

ANOVA: analysis of variance
BMI: body mass index
BML: bone marrow lesion
BML 1: bone marrow lesion detected using the fast spin-echo proton density-weighted sequence only, with absent signal on T1-weighted sequence in the same area
BML 2: bone marrow lesions detected by both fast spin-echo proton density-weighted and T1 sequences
BS: bone surface
BV: bone volume
cl.pores: closed pores
ES: erosion surface
FS: fat suppressed
H&E: hematoxylin and eosin
ICC: intraclass correlation coefficient
K&L: Kellgren and Lawrence
micro-CT: micro-computed tomography
MRI: magnetic resonance imaging
OA: osteoarthritis
OARSI: Osteoarthritis Research Society International
OCU: osteochondral unit
OS: osteoid surface
O.Th: osteoid thickness
OV: osteoid volume
PDFS: fast spin-echo proton density-weighted
ROI: region of interest
SMI: structural model index
Tb.N: trabecular number
Tb.Sp: trabecular separation
Tb.Th: trabecular thickness
TP: tibial plateau
TV: tissue volume
